# Sleep and weight loss in low-income overweight or obese postpartum women

**DOI:** 10.1186/s40608-019-0236-6

**Published:** 2019-04-01

**Authors:** Mei-Wei Chang, Alai Tan, Jonathan Schaffir, Duane T. Wegener

**Affiliations:** 1 College of Nursing, The Ohio State University, 1585 Neil Avenue, Columbus, OH 43210 USA; 2 Department of Obstetrics & Gynecology, The Ohio State University, 370 W. 9th Avenue Columbus, Columbus, OH 43210 USA; 3 Department of Psychology, The Ohio State University, 1835 Neil Avenue, Columbus, OH 43210 USA

**Keywords:** Low-income women, Sleep, Obesity, Postpartum

## Abstract

**Background:**

We conducted secondary data analyses to examine the associations between sleep duration, sleep quality, sleep disturbance and ≥ 5% of weight loss in low-income overweight or obese postpartum women enrolled in a community-based lifestyle behavior intervention study aimed at prevention of weight gain.

**Methods:**

Participants were recruited from the Special Supplemental Nutrition Program for Women, Infants, and Children in Michigan. The Pittsburgh Sleep Quality Index was used to assess sleep duration, sleep quality, and sleep disturbance. All participants were assessed and weighed at baseline (T1, 569 participants), 4-month (T2, 367 participants), and 7-month from T1 (T3, 332 participants). Descriptive statistics and mixed-effects regression analysis were performed.

**Results:**

Participants reported longer sleep duration (*p* = 0.048), better sleep quality (*p* = 0.003) and less sleep disturbance (*p* < 0.001) over time. There were no significant mean body weight changes at T2 and T3. However, a significantly higher proportion of women lost ≥5% of body weight at T3 (23.1%) than T2 (12.5%, *p* = 0.001). Sleep duration, quality, and disturbance were not significantly associated with ≥5% of weight loss.

**Conclusion:**

Improvements in sleep duration, sleep quality and sleep disturbance over time were not associated with ≥5% of weight loss in low-income overweight or obese postpartum women.

**Trial registration:**

Clinical Trials NCT01839708; retrospectively registered February 28, 2013.

## Background

The prevalence of obesity in American women has significantly increased between 2005 and 2014 [1]. One age group contributing to this significant linear trend is women at child-bearing age (young women), especially for those who are low-income [2]. Approximately 50% of young, low-income women are overweight or obese prior to becoming pregnant [3]. These women are at a high risk for persistent obesity and major weight gain later in life, most likely related to excessive gestational weight gain (gaining more than the Institute of Medicine pregnancy weight gain guideline) [4] and significant postpartum weight retention (retaining at least 22 lbs. at 1-year postpartum) [5].

Weight gain of as little as 11 lbs. during young adulthood increases risk of overweight- or obesity-related cancer [6], type 2 diabetes, cardiovascular disease, and nontraumatic death [7]. Also, there is a strong linear association between longer duration of being overweight or obese and incidence of obesity-related cancer [8], all-cause mortality [9], cardiovascular disease [10], and type 2 diabetes [11]. Therefore, it is critically important to help young adults to lose weight. Evidence is clear that even a loss of ≥5% of weight reduces risk of cardiovascular diseases (e.g., by improving glucose, insulin, triglyceride [12, 13], systolic and diastolic blood pressure, HDL cholesterol) [13] and cancer mortality [14].

Identification of risk factors associated with weight gain may potentially inform weight loss intervention efforts to reduce the ongoing obesity epidemic and improve public health. Derangement of sleep is one such risk factor suggested by prior studies, which may be further characterized by problems with sleep duration, sleep quality, or sleep disturbance. The association between short sleep duration and weight gain has recently received increased attention in prospective longitudinal studies. A recent meta-analysis that included 11 prospective cohort studies in adults aged 18 to 72 years found that short sleep duration was associated with weight gain [15]. Consistent with results of the meta-analysis, a systematic review that included 3 prospective cohort studies and 1 cross-sectional study reported that short sleep duration was associated with postpartum weight retention [16]. Also, three other prospective cohort studies consistently reported the association between short sleep duration and weight gain in adults [17–19]. Given the growing evidence that short sleep duration is a potential risk factor for weight gain in adults, it seems logical to examine the association between sleep duration and weight loss. However, no prospective cohort studies have yet been conducted in overweight or obese adults. Only two lifestyle behavior weight loss intervention studies for middle-aged overweight or obese adults have examined such association. These studies have consistently shown that increased sleep duration was associated with weight loss [20, 21]. However, it is unknown whether the positive results found in middle-aged overweight or obese adults could be applied to low-income overweight or obese postpartum women.

The association between sleep quality and weight loss has also received little attention, with only three weight loss intervention studies for middle-aged overweight or obese adults investigating this association. These studies have yielded mixed results [20–22]. While two studies reported that improved sleep quality was associated with successful weight loss at 3–4 months [20] and at 6 months from baseline [22], one study did not find such association at the end of a 6-month intervention or at 12-month and 18-month follow ups [21]. Few longitudinal prospective studies have investigated an association between sleep disturbance and weight loss in overweight or obese adults. These studies have consistently reported no association between sleep disturbance and weight loss in middle-aged women who enrolled in a weight loss intervention study [23] and in adults enrolled in a 30-year prospective cohort study [24].

Sleep duration, sleep quality, and sleep disturbance are potentially modifiable risk factors for obesity. However, only a limited number of studies have investigated the associations between sleep duration, sleep quality, sleep disturbance and weight loss in overweight or obese adults. Also, these studies mainly focused on middle-aged obese adults. Our review of literature suggests a knowledge gap in these associations for low-income overweight or obese postpartum women. To design weight loss intervention studies for low-income overweight or obese postpartum women, it is critically important to understand the association between sleep and weight loss. The objective of this secondary data analysis was to examine the associations between sleep duration, sleep quality, sleep disturbance and ≥ 5% of weight loss in low-income overweight or obese postpartum women enrolled in a community-based lifestyle behavior intervention study aimed at prevention of weight gain. Although sleep was not a target of the behavior intervention, examining such associations may still be informative for designing future interventions in this population.

## Methods

Participants were recruited from the Special Supplemental Nutrition Program for Women, Infants, and Children (WIC) in Michigan between September 2012 and January 2015. WIC is a federally funded program that serves low-income pregnant, breastfeeding and postpartum women and children (0–5 years) in the US. To be eligible to participate in this study, women had to be non-pregnant, 18–39 years old, between 6 weeks and 4.5 years postpartum, non-Hispanic Black (Black) or White (White), free of type 1 or 2 diabetes, and overweight or obese (body mass index [BMI] between 25.0 and 39.9 kg/m^2^). Height and weight were measured during recruitment and were used to calculate BMI. Details about the study recruitment and eligibility have been previously described [25]. The study procedure was approved and monitored by Michigan Department of Health and Human Services and Michigan State University Institutional Review Boards.

### Randomization and intervention

A more complete description of the community-based lifestyle behavior intervention study aimed at prevention of weight gain has been previously published [26]. Briefly, participants (*N* = 569) were randomized in a 2:1 ratio to an intervention (*n* = 387) or comparison group (*n* = 182). During the 16-week intervention, the intervention group viewed 10 intervention videos at home: a video every week (weeks 1–4) followed by every other week (weeks 6–16). The video topics did not include any contents related to sleep but included stress management (4 videos), healthy eating (5 videos), and physical activity (1 video). Intervention participants also dialed in to 10 peer support group teleconferences led by community peer educators and WIC professionals who were trained in motivational interviewing and group facilitation. The comparison group received printed educational messages on stress management, healthy eating, and physical activity and no contents related to sleep. We assessed all participants’ body weight (in person, primary outcome) at baseline (T1), immediately after the 16-week intervention (T2, 4 months from T1), and at a 3-month follow up (T3, 7 months from T1). We did not detect any significant differences between the intervention and comparison groups in mean body weight at T2 or T3 [27]. Prior to conducting this secondary analysis study, we investigated whether there were significant differences in sleep duration, sleep quality, and sleep disturbance between the intervention and comparison groups. Since we did not find any differences between groups, in this paper we presented results that combined all study participants regardless of their group assignments.

### Measurements

#### Demographics

Participants self-reported their birthday (to calculate age) and their youngest child’s birthday (to calculate postpartum period). They also provided information on race/ethnicity, smoking, education, employment, and current breastfeeding status (yes/no).

#### Body weight

Participants returned to the WIC office where they were recruited to have body weight measured. Body weight was measured using an electronic digital scale (Seca 869, Germany) to the nearest 0.2 lbs. while participants wore light clothing and no shoes.

#### Sleep duration, sleep quality, and sleep disturbance

We used subscales from the Pittsburgh Sleep Quality Index, which has established validity and reliability for each subscale [28], to measure sleep duration, quality and disturbance. Participants were instructed to provide responses that reflected the majority of days and nights in the past month. For sleep duration (1 item), participants were asked “How many hours of actual sleep did you get? This may be different from the number of hours you spend in bed.” Response options range from more than 7 h (0), between 6 and 7 h (1), between 5 and 6 h (2), and less than 5 h (3). For sleep quality (1 item), they were asked to rate their overall sleep quality. Response options range from very good (0) to very bad (3). For sleep disturbance (9 items), participants were asked to respond to a list of questions asking about factors contributing to their trouble sleeping. Response options range from none in the last month (0) to three or more times a week (3). The overall sleep disturbance score was the mean of the 9 responses. A higher score indicates more sleep disturbance.

### Statistical analysis

Descriptive statistics were used to summarize the sample at each time point on baseline characteristics and outcomes, including sleep duration, sleep quality, sleep disturbance, and weight outcomes (weight in pounds, % weight change from T1, and whether or not achieving ≥5% weight loss from T1). We compared the baseline demographic characteristics across time (T1 to T3). We also examined the overall trajectories of outcomes across time using mixed-effects logistic regression for dichotomized outcomes (sleep duration, ≥ 7 h/per night [adequate or longer sleep duration] vs. < 7 h/per night [shorter sleep duration]; ≥ 5% weight loss from baseline, yes vs. no) and mixed-effects linear regression for continuous outcomes (sleep quality, sleep disturbance, body weight, and weight change from baseline – absolute (or mean) change and % change). We then examined whether weight change across time was related to sleep. As the ≥5% weight loss from baseline was the only weight outcome that had significant change across time, we compared women with < 5% weight loss to those with ≥5% weight loss from baseline in sleep duration, sleep quality, and sleep disturbance. Chi-square statistics were performed for categorical variables (sleep duration) and two-sample t-tests were applied for continuous variables (sleep quality and sleep disturbance). We used SAS 9.4® (SAS Institute, Cary, NC) for the statistical analysis. All tests were two-sided at a significance level of 0.05.

## Results

Table 1 presents demographic characteristics of the study cohort at each time point of data collection. At baseline, the mean age of participants was 28.5 ± 5.03 years, and the mean time from last delivery was 1.72 ± 1.28 years. Approximately 79% of participants were non-Hispanic White; 67% had at least some college education; 46% were employed part-or-full time. Also, most participants did not breastfeed (84%), were non-smokers (74%) and were obese (64%). In spite of sample attrition (T1 = 569 participants, T2 = 367 participants, and T3 = 332 participants), there were no significant differences in demographic characteristics across the 3 time points.

**Table 1:** Demographic Characteristics of Low-Income Overweight or Obese Postpartum Women

Characteristics	Mean ± SD or N (%)			P-value^$^
	T1 (*N* = 569)	T2 (*N* = 367)	T3 (*N* = 332)	
Age (years)	28.54 ± 5.03	29.17 ± 4.91	29.42 ± 4.86	1.00
Postpartum period (years)	1.72 ± 1.28	1.73 ± 1.27	1.74 ± 1.28	0.98
Race				
Black	121 (21.27)	68 (18.53)	63 (18.98)	0.53
White	448 (78.73)	299 (81.47)	269 (81.02)	
Education				
High School or less	187 (32.86)	116 (31.61)	102 (30.72)	0.80
At least some college	382 (67.14)	251 (68.39)	230 (69.28)	
Employment				
Employed (FT/PT/Self)	262 (46.05)	161 (43.87)	139 (41.87)	0.66
Unemployed	118 (20.74)	66 (17.98)	61 (18.37)	
Other (homemaker/student/other)	189 (33.22)	140 (38.15)	132 (39.76)	
Current smoker				
No	421 (73.99)	296 (80.65)	260 (78.31)	0.12
Yes	148 (26.01)	71 (19.35)	72 (21.69)	
Current breastfeeding				
No	475 (83.48)	290 (79.02)	267 (80.42)	0.76
Yes	94 (16.52)	77 (20.98)	65 (19.58)	
Body mass index (BMI)	32.04 ± 4.29	31.77 ± 4.35	31.60 ± 4.30	1.00
BMI category				
Overweight (BMI 25–29.9 kg/m^2^)	205 (36.03)	142 (38.69)	131 (39.46)	0.83
Obese I (BMI 30–34.9 kg/m^2^)	209 (36.73)	131 (35.69)	122 (36.75)	
Obese II (BMI 35.0–39.9 kg/m^2^)	155 (27.24)	94 (25.61)	79 (23.80)	
Randomization				
Intervention group	387 (68.01)	236 (64.31)	213 (64.16)	0.80
Comparison group	182 (31.99)	131 (35.69)	119 (35.84)	

T1: baseline, T2: 4 months from T1, T3: 7 months from T1. FT: employed full-time, PT: employed part-time, self = self-employed

^$^*P*-values were derived using mixed-effects linear regression modeling for continuous variables (age, postpartum period, BMI) and mixed-effects logistic regression for categorical variables (race, education, employment, current smoker, current breastfeeding, BMI category, and randomization). We did not present coefficient or odds ratio estimates from the regression models since the descriptive statistics (Mean ± SD and %) in the table are more straightforward to illustrate the extent of change across time

Table 2 shows change in sleep duration, sleep quality, sleep disturbance, and body weight (mean and % change) over time. Participants reported significant improvements in sleep duration (*p* = 0.048), sleep quality (*p* = 0.003) and sleep disturbance over time (*p* < 0.001). Although there were no significant changes in mean body weight across the 3 time points, there was a trend of decreasing mean body weight over time. A significantly higher proportion of participants lost ≥5% of body weight at T3 (23.1%) than T2 (12.5%; *p* = 0.001).

**Table 2:** Change in Sleep Duration, Sleep Quality, Sleep Disturbance, and Body Weight across Time

	Mean ± SD or N (%)			P-value^$^
	T1 (*N* = 569)	T2 (*N* = 367)	T3 (*N* = 332)	
Sleep Duration				
< 7 h	457 (80.32%)	249 (73.67%)	230 (73.95%)	0.048
≥ 7 h	112 (19.68)	89 (26.33%)	81 (26.05%)	
Sleep quality^a^	1.44 ± 0.78	1.35 ± 0.82	1.30 ± 0.75	0.003
Sleep disturbance^b^	1.42 ± 0.58	1.26 ± 0.67	1.21 ± 0.67	< 0.001
Weight	190.8 ± 29.85	188.5 ± 31.22	187.9 ± 32.07	0.389
Weight change from T1	n/a	−.08 ± 9.72	−.72 ± 12.46	0.218
% Weight change from T1	n/a	−0.1% ± 5.1%	−0.4% ± 6.7%	0.253
Having ≥ 5% weight loss from T1				
No	n/a	300 (87.4%)	246 (76.9%)	0.001
Yes	n/a	43 (12.5%)	74 (23.1%)	

T1: baseline, T2: 4 months from T1, T3: 7 months from T1. ^a^The lower score, the better sleep quality. ^b^The higher score, the more sleep disturbance

^$^P-values were derived using mixed-effects linear regression modeling for continuous variables (sleep quality, sleep disturbance, weight, weight change from T1, and % weight change from T1) and using mixed-effects logistic regression for categorical variables (sleep duration and having ≥5% weight loss from T1). We did not present coefficient or odds ratio estimates from the regression models since the descriptive statistics (Mean ± SD and %) in the table are more straightforward to illustrate the extent of change across time

Table 3 presents results of comparison between women with < 5% and those with ≥5% weight loss from baseline by sleep duration, sleep quality and sleep disturbance. Improvements in sleep duration, sleep quality and sleep disturbance were not associated with ≥5% of weight loss at T2 or T3.

**Table 3:** Comparison of Women with < 5% Vs. ≥ 5% Weight Loss from Baseline by Sleep Duration, Sleep Quality, and Sleep Disturbance

	Weight loss, T2 vs. T1			Weight loss, T3 vs. T1		
	< 5%	≥ 5%	P-value^$^	< 5%	≥ 5%	P-value^$^
	*N (row %)*			*N (row %)*		
Sleep duration						
Baseline						
≥ 7 h	61 (85.92)	10 (14.08)	0.658	50 (73.53)	18 (26.47)	0.819
< 7 h	239 (87.87)	33 (12.13)		196 (77.78)	56 (22.22)	
Follow-up						
≥ 7 h	78 (88.64)	10 (11.36)	0.852	63 (79.75)	16 (20.25)	0.288
< 7 h	203 (87.88)	28 (12.12)		168 (75.00)	56 (25.00)	
Change from baseline						
Kept ≥7 h	36 (87.80)	5 (12.20)	0.783	31 (79.49)	8 (20.51)	0.184
Increased to ≥7 h	42 (89.36)	5 (10.64)		32 (80.00)	8 (20.00)	
Kept < 7 h	183 (87.98)	25 (12.02)		153 (76.50)	47 (23.50)	
Decreased to < 7 h	20 (86.96)	3 (13.04)		15 (62.50)	9 (37.50)	
	*Mean ± SD*			*Mean ± SD*		
Sleep quality^a^						
Baseline	1.42 ± 0.75	1.40 ± 0.85	0.822	1.44 ± 0.74	1.46 ± 0.83	0.871
Follow-up	1.35 ± 0.81	1.16 ± 0.75	0.171	1.32 ± 0.77	1.24 ± 0.72	0.388
Change from T1	−0.07 ± 0.85	−0.29 ± 0.65	0.131	−0.10 ± 0.82	− 0.24 ± 0.76	0.224
Sleep disturbance^b^						
Baseline	1.41 ± 0.58	1.44 ± 0.59	0.764	1.40 ± 0.58	1.49 ± 0.58	0.277
Follow-up	1.27 ± 0.65	1.23 ± 0.68	0.724	1.21 ± 0.67	1.28 ± 0.63	0.411
Change from T1	−0.14 ± 0.70	−0.21 ± 0.51	0.458	−0.19 ± 0.72	−0.20 ± 0.55	0.883

T1: baseline, T2: 4 months from T1, T3: 7 months from T1. ^a^The lower score, the better sleep quality. ^b^The higher score, the more sleep disturbance

^$^P-values were derived using Chi-square statistics for categorical variables (sleep duration) and two-sample t-tests for continuous variables (sleep quality and sleep disturbance)

## Discussion

This is the first study to examine the associations between sleep duration, sleep quality, sleep disturbance, and ≥ 5% weight loss in low-income overweight or obese postpartum women using prospective longitudinal data. We observed that improvements in sleep duration, sleep quality, and sleep disturbance over time (T2 and T3) were not associated with ≥5% of weight loss. One possible explanation of the overall non-significant findings is related to the low magnitude of changes in sleep duration, sleep quality, and sleep disturbance. This means that though overall amount of sleep duration, sleep quality, and sleep disturbance improved significantly over time, the changes were relatively small; and sleep quality and sleep disturbances remained poor. Also, the overall improvement in sleep might have been impacted by our intervention that included stress management. A recent study of pregnant women has reported that higher levels of stress were associated with poor sleep [29]. In our intervention study, we found that the intervention group had a significant reduction in stress at T2 and the positive change remained at T3. However, our results also revealed that the comparison group had a significant reduction in stress at T3 [30]. Finally, it is possible that as children grow older, they might sleep better [31] and require less attention from their caregivers at night. Also, they might sleep apart from their mothers, which was associated with better sleep in mothers [32].

Our finding showing no association between sleep duration and ≥ 5% weight loss is inconsistent with findings of a previous weight loss intervention study [22]. The inconsistency might have related to the study sample. Whereas we focused on postpartum women whose sleep is often dependent on different factors (e.g., children’s sleep), the prior studies focused on middle-aged adults. Similarly, our findings on sleep quality and weight loss were consistent with one study of middle-aged adults [23], but not another study of middle-aged adults [20]. We are unable to explain the discrepancy. Finally, our finding indicating no association between sleep disturbance and weight loss was consistent with two prior studies [23, 24].

There are strengths of the present research. Our findings were based on analysis of a prospective longitudinal dataset that included a homogeneous sample of low-income overweight or obese postpartum women. Also, body weight was measured in person rather than based on self-report. Although sleep was not targeted as a behavior for intervention in the intervention study, we used validated instruments to assess sleep duration, sleep quality, and sleep disturbance that appropriately measure these characteristics as they occur in this population. Even so, several limitations of the study need to be considered when interpreting our findings. First, this was a secondary data analysis. The level of power to detect any significant differences in the measurements that we chose is unknown but could be lower than desired. Second, we used self-reported sleep data over an extended period of time (the past month). Though these measures have proven helpful in predicting weight loss in some studies, asking respondents to report sleep duration, sleep quality, and sleep disturbance over an extended period could lead to biases in over-emphasizing recent experience rather than actually characterizing the sleep experience over the requested period. If recent experiences are more variable than the requested trends in sleep experience over time, a measure bias toward recent experience could produce weaker associations with longer-term outcomes such as weight loss than would a measure that more faithfully captures the longer-term trend in sleep experience. Third, the dropout rate across each time point of the study was substantial, though the demographic characteristics of the sample remained consistent over each time point of study data collection. Fourth, the length of the study may have been too short to demonstrate clinically significant results. Finally, the mean changes in weight and sleep scores were small, even when statistically significant. It is possible that larger amounts of weight loss may be observed with larger changes in sleep behaviors.

## Conclusions

Our findings show that improvements in sleep duration, sleep quality, and sleep disturbance were not associated with ≥5% of weight loss from baseline in low-income overweight or obese postpartum women. Future prospective studies of the target population may be helpful to identify mediators and moderators that affect the association between sleep and weight loss.

## Abbreviations

BMI: Body Mass Index
WIC: The Special Supplemental Nutrition Program for Women, Infants, and Children.
